# Formal Education in Leadership and Management for the Practicing Clinician

**DOI:** 10.5041/RMMJ.10561

**Published:** 2025-12-15

**Authors:** Richard H. Savel

**Affiliations:** 1 Chair, Department of Medicine, Jersey City Medical Center, Jersey City, New Jersey, USA; 2 Clinical Professor of Medicine, Rutgers New Jersey Medical School, Newark, New Jersey, USA

**Keywords:** Administration, budgeting, finance, leadership, management, strategy

## Abstract

As clinicians progress in their careers, they are often tasked with projects and responsibilities that require additional education, knowledge, and training in leadership and management. In the past, they were expected to pick up these skills along the way; current expectations, however, are different. Today, several avenues are available to clinicians to acquire and refine these competencies. This narrative review provides a structured overview of the education and training available—both with and without formal credentials—and outlines potential opportunities and pathways for developing leadership and management skills, particularly among critical care physicians.

## INTRODUCTION

The education of clinicians includes formal training in how to rapidly assess, evaluate, and manage patients. Their studies are demanding, and candidates must pass numerous examinations before becoming legally qualified and able to safely manage complex clinical situations unsupervised. But as the clinician’s career progresses, many are tasked with projects for which they have not been trained and are made accountable for outcomes over which they have little or no control. These administrative/leadership roles are just as important and challenging as their clinical responsibilities; examples include being made medical or nursing director of a unit, being responsible for managing other clinicians, or becoming accountable for clinical and safety/quality outcomes for multiple units. When successfully demonstrating proficiencies in these areas, such clinicians can find themselves with new responsibilities that are often outside their original primary area of specialization (e.g. nursing, pharmacy, medicine, surgery, or anesthesia). In today’s world, proficiency in these skills can no longer be attained along the way. For clinician-leaders and administrators to have a “seat at the table” when important organizational decisions are being made, they are expected to speak the language of leadership and management with its own vocabulary, education, qualifications, and credentials. Hence, this narrative review introduces the reader to the rationale and practical aspects of formal education in leadership and management, providing guidance to critical care practitioners, no matter where they may be in their leadership journey. This article should be relevant to critical care clinicians currently in training (who may be contemplating an interest in leadership/management), recent graduates/junior faculty (where they may have found themselves taking on more administrative responsibility without even realizing it), or mid-career clinicians (who perhaps are considering a switch to a primarily administrative/leadership role but may not have the requisite credentials to be a competitive candidate).

Despite the importance of administrative, management, and leadership skills for the average clinician, time is limited and not all practitioners may need or want this additional education. For example, in the critical care specialty, the American Board of Internal Medicine Critical Care Medicine Examination Blueprint^1^ states that (only) 2% of the exam is devoted to “research, administration, and ethics” combined,^2^ and the Accreditation Council for Graduate Medical Education program requirements for critical care medicine^3^ have no explicit mandate for integration of leadership styles or management techniques. Nevertheless, critical care clinicians who want to expand their administrative, management, and leadership skillset should have access to a readily accessible, easily digestible document to help guide them through the process.

## LEADERSHIP AND MANAGEMENT IN THE CONTEXT OF ORGANIZATIONAL BEHAVIOR

### Leadership

Though the concept of leadership is a somewhat nebulous term,^4^ an agreed-upon textbook definition of leadership is “the ability to influence a group toward the achievement of a vision or a set of goals.”^5^(p One of the most important lessons to be learned from the field of organizational behavior is that leadership is a skill that can be taught.^6^,^7^ Importantly, there are different formal leadership styles, and leaders can and should select elements from each that align with their own personality traits and strengths, rather than feeling they must change their core identity as they advance in their leadership journey. Some examples of the most common leadership styles are authentic, authoritative (versus authoritarian), transformational, and servant (Table 1).^8^–^11^

**Table 1:** Leadership Styles with Definitions.^12^,^13^

Leadership Style	Definition	Strengths/Limitations	Commentary
Authentic	Emphasizes honesty, ethical conduct, and openness. Leaders share information transparently and invite input from team members to guide decision-making.	Strength: Builds trust and credibility through transparency. Limitation: Excessive openness may occasionally be counterproductive.	Modern, values-driven style; well aligned with current healthcare expectations.
Authoritarian	Characterized by *centralized control*; the leader retains nearly all authority and directs others with minimal collaboration; often described as coercive or command-based.	Strength: May be effective in crises demanding rapid, unambiguous direction. Limitation: Suppresses autonomy; may reduce morale or innovation.	Generally discouraged in healthcare settings where collaboration is key.
Authoritative	Differs from Authoritarian in that the focus is on *mentoring* and *guiding* teams toward a shared vision. The leader defines goals clearly, anticipates challenges, and provides structured support to achieve objectives.	Strength: Clarifies direction and expectations. Limitation: May unintentionally limit upward communication or feedback.	Appropriate when clear guidance is required; context-dependent utility.
Transformational	Motivates team members to exceed baseline expectations by uniting them around an inspiring vision and sense of purpose.	Strength: Enhances motivation, engagement, and integrity. Limitation: Long-term focus may slow short-term decision-making.	Highly relevant in modern organizations; widely regarded as an effective framework for change leadership.
Servant	Centers on supporting and empowering others rather than exercising authority; prioritizes the growth, well-being, and success of the team.	Strength: Builds loyalty, shared purpose, and respect. Limitation: Less effective in situations requiring swift, top-down action; may need cultural adaptation.	Particularly compatible with healthcare values emphasizing service and compassion.

A combination of transformational leadership^9^,^12^— where the focus is on inspiration rather than command—and servant leadership is frequently cited as an effective approach. In servant leadership, a term coined by Robert K. Greenleaf in a 1970 essay,^13^ the primary role of the leader is to support their followers (Table 2). Servant leadership inverts the norm: instead of the people working to serve the leader, the leader exists to serve the people. A servant leader is a leader among equals, a crucial point for healthcare leaders, as they frequently have the challenge of leading their former peers. According to Greenleaf, a Servant Leader should be focused on asking if those served grow as persons. Do they, while being served, become healthier, wiser, freer, more autonomous, and more likely themselves to become servants?^10^,^13^–^16^ An example of this type of leadership was the theme of Marquet’s work, *Turn the Ship Around*, describing an empowering and supportive approach to managing subordinates.^17^ For a critical care clinician who either has or plans to assume more leadership roles, taking some time to delve deeper into the literature of leadership styles is time well spent: the more of a structured approach one has to a situation, the less likely one is to come up with reflexive responses that one may regret in the future.

**Table 2:** Characteristics of an Effective Servant-minded Organization.^13^,^15^,^16^

Characteristic	Description
Values People	Believes, serves, and non-judgmentally listens to others
Builds Community	Develops strong collaborative and personal relationships
Develops People	Provides encouragement, affirmation, and opportunities for learning and growth
Displays Authenticity	Is open, accountable, and willing to learn from others
Provides Leadership	Foresees the future, takes initiative, and establishes goals
Shares Leadership	Facilitates and shares power

### Management

Though the concepts of leadership and management are both important, they are not synonymous (Table 3). As noted above, the goal and role of leadership/leaders is to use strategy to develop short-term and long-term plans as well as create an organizational culture and inspire members of the organization toward a unified goal. In contradistinction, the primary focus of management is to work with front-line staff to operationalize the ideas and vision of the leadership team.^12^,^18^–^22^ Leadership takes a *what-should-we-do* approach, whereas management takes a *how-do-we-do-it* approach. An example of leadership in healthcare would be to recognize that Intensive Care Unit patients need world-class care with a target of zero central line infections and urinary tract infections. The management perspective on the same issue would be the creation of a dedicated committee devoted to achieving this goal by tracking the rates of these infections and designating certain staff members to interface with the clinical teams to heighten awareness of these hospital-acquired conditions, thereby creating a structured approach to prevention. It is common for clinician leaders (especially in smaller or resource-constrained organizations) to simultaneously perform leadership and management functions or to frequently alternate between the two states.

**Table 3:** Characteristics of Leadership, Management, and Both.^19^,^20^,^22^

Management	Both	Leadership
Planning	Communicating	Influencing
Organizing	Problem-solving	Motivating
Staffing	Decision-making	Inspiring
Coordinating		Creating
Controlling		Mentoring

In addition to simply understanding the definition and meaning of the concepts of leadership and management, it is often the case that for one to become a successful leader in healthcare, one must master certain management skills that are not part of routine training. A commonly used example is that of how to run an effective meeting^23^ (e.g. creating and archiving agendas and minutes as well as understanding and implementing the organizational behavior of groups—allowing everyone to provide input while preventing disruptions from attendees who may be more comfortable speaking in groups). Though at first glance these ideas may sound simple—and potentially even trivial—the determination of the optimal frequency and size of meetings can be complex and have significant consequences for the efficiency and functionality of an organization.^24^

### Other Important Aspects of Organizational Behavior

Over and above leadership and management, there are numerous other important relevant concepts that fall under the rubric of organizational behavior.^25^ As discipline, organizational behavior is a multidisciplinary study—with contributions from the fields of psychology, social psychology, sociology, and anthropology—focused on employee interactions and organizational processes that promote more efficient and cohesive organizations. Examples include: motivation, personality types, organizational culture, group behavior, decision-making, understanding teams (groups versus teams),^26^ communication, power/politics/influence, conflict management,^27^–^30^ the art of negotiation,^31^–^33^ organizational design, and emotional intelligence.^34^

### Accounting, Finance, and Budgets

Potentially daunting areas of leadership and management that clinicians may encounter as they progress further into their leadership journey can be the topics of accounting, finance, and budgets, with a significant number of us having little or no formal training in this area.^35^–^37^ Firstly, it must be noted that the field of accounting is broadly divided into financial and managerial accounting.^38^,^39^ Financial accounting involves recording, summarizing, and reporting transactions and economic activity. In contrast, managerial accounting identifies, measures, analyzes, interprets, and communicates financial information to internally support organizational goals.^39^ Financial accounting is a highly regulated industry where corporations are mandated to present their financial transactions externally (e.g. to shareholders, lenders, banks, and government agencies). It is beyond the scope of this paper to provide significant detail for either financial or managerial accounting other than to say that the customary financial statements (e.g. balance sheets, income statements, statements of shareholder equity, and statement of cash flow) are largely standardized and agreed-upon in terms of their content and format. Such rules do not apply to managerial accounting as the data are used for internal organizational purposes. Remember that a budget is simply a predicted income statement/profit and loss statement. Partnering with a local organizational administrative/finance asset can help to facilitate the generation of relevant budgets. Though it is not mandatory that a formal degree be obtained to develop familiarity and a comfort level regarding the financial statements, some form of education may be of significant value as the clinician leader develops in their journey.

## TRANSLATING IDEAS TO REALITY

### The Basics of a Business Case (Plan)

Clinician leaders will inevitably want to obtain a product or a service to enhance some component of their unit, division, or department. When they go to their superior and ask for such an item, they will be asked to provide financial justification, the “return on investment” or ROI. In other words, they will be asked to provide a business case. This is sometimes referred to as a business plan,^40^–^43^ though the concept of a business plan is usually of a much larger scope than a business case where a particular item, or project, is being asked about rather than a document that emphasizes the entire business model. Regardless of whether it is called a business case or business plan, the purpose of this document is to serve as a summary of the project, the capital item, the service, or additional personnel that are being requested, with an associated financial justification. Though there are numerous different formats for the business case, one recommended format can be seen in Table 4.^41^ It begins with an *executive summary*, a brief description of the overall plan including a key-point summary of the entire document. Next, there is an *overview* and *introduction* followed by *assumptions and rationale* with a subsequent *project summary*. The next three sections—*financial analysis*, *benefits and business impacts*, and *schedule with milestones*—are rather quantitative and often will require the assistance of a financial asset of the organization to be properly completed. This is where one would place the formal ROI section of the business case. The last three sections are *risk and contingency analysis*, *conclusion and recommendations*, as well as any *appendices*. It is important to note that it is certainly not mandated that all the sections be present in every business case. The idea with this particular format is to help provide a structured approach when requesting additional resources whether they be capital expenses, services, or personnel. Finally, we would add that in the healthcare industry there may be cases where the ROI may not be purely financial.

**Table 4:** The Basics of a Business Case.^41^

Section Heading	Description
1. Executive Overview	A brief synopsis (usually one to two pages) that captures the proposal’s purpose, central recommendations, and most important conclusions.
2. Background and Context	Establishes the organizational setting, explains the current circumstances, outlines the challenge or opportunity, and introduces the proposed direction.
3. Foundational Logic and Key Assumptions	Specifies the guiding conditions and justifications for the proposal—such as staffing needs, regulatory influences, marketplace trends, or financial drivers.
4. Project Outline and Objectives	Provides a thorough summary of the initiative, defining its aims, scope, required resources, timeline, responsible personnel, indicators of success, and projected results.
5. Fiscal Analysis and Budget Review	Describes the financial dimension of the proposal, including estimated expenses, anticipated savings or revenue, ROI calculations, and fiscal assumptions. May feature models or projections.
6. Anticipated Benefits and Organizational Value	Explains the expected gains—both measurable and intangible—such as improved performance, innovation, efficiency, or team engagement.
7. Implementation Timeline and Key Milestones	Lays out the schedule for execution, identifying major phases, benchmarks, and deliverables. Communication or rollout plans may be integrated here.
8. Risk Evaluation and Backup Strategies	Identifies possible obstacles or uncertainties and defines how these will be addressed to ensure continued progress. May include scenario or dependency analyses.
9. Summary Conclusions and Action Guidance	Consolidates the major findings and provides a clear statement of why the initiative should move forward.
10 Supporting Documentation	Contains additional materials—financial data, detailed analyses, or references—that reinforce the main content of the proposal.

ROI, return on investment.

### Understanding Operations Management: Supply Chains and Project Management

The final discipline to consider from an administrative perspective is operations management. Operations management is the administration of business practices to create the highest level of efficiency possible within an organization. It is concerned with converting materials and labor into goods and services as efficiently as possible to maximize the profit of an organization.^44^–^47^ Some of the relevant terms and ideas that come under the heading of operations management include *supply chains*, *processes*, and *project management*. During and post-COVID, supply chain failures underscored how operational issues directly impact clinical care, reinforcing the relevance of operations management for healthcare leaders.

Many organizations use formal project management methodologies—such as Lean, Six Sigma, or other structured frameworks—to coordinate complex projects across multiple departments. It is beyond the scope of this review to provide an in-depth discussion of the field of operations management. However, it is important to understand that larger organizations often have a project management office where project managers interface with clinician leaders to help implement and operationalize projects. These initiatives are frequently complex, span multiple clinical departments, and require project management to ensure efficient execution.^48^–^51^

## STRATEGY: THE BIGGER VIEW

### What Is Strategy and Why Is It Important?

**Figure 1:**
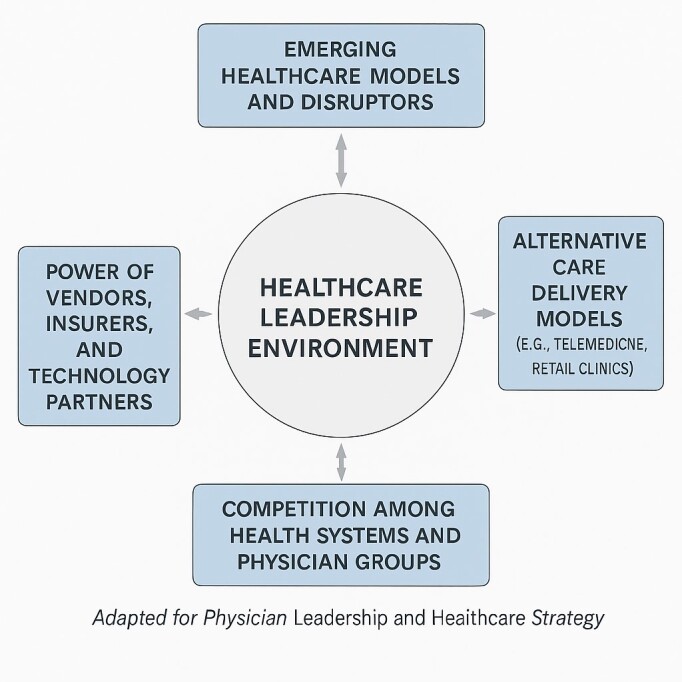
Porter’s Five Forces Adapted for Physician Leadership and Healthcare Strategy.^52^,^57^

Though there are many definitions of strategy, one definition is that strategy is an integrated overarching concept of how a business will achieve its objectives.^52^ Strategy is an area where clinicians often have no training whatsoever as this topic is not truly relevant to the practicing clinician.^53^–^56^ Clinicians, particularly those in critical care, are trained to focus on the patient directly in front of them. Strategic thinking is fundamentally different—and often runs counter to—the routine practice, training, and educational focus of critical care clinicians. The strategic goal is to answer questions related to organizational performance in five years and the resources needed to get there. A commonly used term when discussing strategy is competitive advantage, defined as anything that gives a company an edge over its competitors, helping it attract more customers and grow its market share.^52^ One of the important paradigms used when learning about strategy is the concept of Porter’s five forces created by Harvard Business School Professor Michael E. Porter in 1979.^57^ Figure 1 represents an adaption of Porter’s framework tailored to the healthcare context. Porter’s five forces model can be used to help explain the level of competition in any industry and is often employed when creating corporate strategy. The first force describes how easy it is (or not) for new entrants to join the marketplace. If it is easy to enter the marketplace, there will be significant competition, hence, strategic efforts should be made to limit how easy it is to enter the marketplace. The second force analyzes the rivalry among existing competitors. The third force looks at the threat of substitutes. A classic example outside of medicine is the beverage industry where people can easily substitute one product for another. The fourth force is the bargaining power of industry suppliers. Hospitals must deal with such issues frequently when purchasing products. The fifth and final force is the bargaining power of the consumer. In healthcare, it can be very challenging for the average consumer to switch from one provider to another. It is not as easy as, let us say, switching from one grocery store to another. Overall, the concept of learning about and practicing strategy becomes more important the more the role of the critical care practitioner changes from clinical to administrative.

## THE LOGISTICS FOR OBTAINING THE EDUCATION

### Classwork versus Obtaining Another Degree

Clinicians are free to take as few or as many classes as are necessary depending on how motivated they are to advance in a particular field of expertise. Multiple options are available: taking a single online class to learn the basics of leadership; attending a multi-week university course regarding leadership, management, and finance; and advancing to completion of a formal degree in medical management/leadership. This author greatly benefited from a class given at the Harvard School of Public Health called “Leadership Development for Physicians in Academic Medical Centers.”^58^ This multi-week, in-person class provides an intense learning environment for clinicians, specifically physicians interested in furthering their knowledge and experience regarding leadership and management. The American Association for Physician Leadership has created a “mini-MBA” program called the Certified Physician Executive Program (CPE).^59^ The coursework for this program is usually taken over several months and concludes with a 3.5-day in-person capstone session where the student must present a simulated project to senior leadership and receives formal evaluation and feedback.

### Formal Advanced Degrees in Medical Management with Examples

There is no clear-cut advantage of one advanced degree program over another. This is a very personal and highly subjective decision with no one right answer. Examples of such degrees include: MBA (Master of Business Administration), eMBA (executive MBA), MMM (Master of Medical Management), MHA (Master of Healthcare Administration), MPA (Master of Public Administration), as well as Doctor of Nursing Practice programs in leadership. The usual factors involved regarding which path to choose include: the overall time commitment, the balance of in-person and online coursework, as well as financial issues. Another decision is whether it should be a medically oriented program. Clearly, each student’s needs and goals will differ. Hence, significant exploration into the available programs should be done before making a final decision.

### Example of an MBA Curriculum

For the sake of completeness, Table 5 provides a sample MBA curriculum (approved by the accreditation body for such programs, the Association to Advance Collegiate Schools of Business, AACSB), completed by the author. The core curriculum of 11 classes can be taken in as little as two years, or as long as four years. This example provides a description of the types of classes and educational content that would be received from an MBA program, not including elective courses and certain prerequisites.

**Table 5:** Example of Core Curriculum for a Masters of Business Administration (MBA).

Course Title
Leadership and organizational behavior
Business data analysis and statistical methods
Services marketing management
Operations management
Human resource management
Business law
Economic analysis for managers
Finance and managerial accounting
Corporate finance
Organizational strategy
Strategic information management

## CONCLUSIONS

In conclusion, this narrative review has attempted to provide the reader with a structured approach when contemplating enhancing one’s leadership and management skillset. While the overall focus has been on critical care clinicians, these same approaches can be taken by all members of a multidisciplinary critical care team and beyond. As challenging as leadership and management responsibilities in the healthcare setting may be, obtaining the appropriate education and training has the potential to make the leadership journey less arduous and formidable and more likely to be meaningful, rewarding, and, ultimately, successful.
